# Sometimes There Is More Than One Puzzle on the Table: Pneumococcal Bacteremia as a New Systemic Lupus Erythematosus Presentation

**DOI:** 10.1155/2015/970289

**Published:** 2015-12-20

**Authors:** Fereshte Sheybani, Hamidreza Naderi, Zahra Mirfeizi, Sedigheh Erfani

**Affiliations:** ^1^Department of Infectious Diseases, Faculty of Medicine, Mashhad University of Medical Sciences, Mashhad 91388-13944, Iran; ^2^Department of Rheumatology, Faculty of Medicine, Mashhad University of Medical Sciences, Mashhad 91388-13944, Iran

## Abstract

Infection is common and a leading cause of death in patients with systemic lupus erythematosus (SLE). SLE is associated with a diverse spectrum of immune impairments including humoral defects and hypocomplementemia that contribute to a lupus patient's increased susceptibility to infection with encapsulated bacteria. Nonetheless, there are only few reports of severe invasive bacterial infection as the initial presentation of SLE in the literature. Here, we report a rare case of SLE presenting with pneumococcal bacteremia. Based on the high resolution chest computed tomography and the result of blood cultures, the bacteremia was assumed to be secondary to pneumococcal pneumonia.

## 1. Case Presentation

A 14-year-old girl presented to the emergency department complaining of fever and bilateral knee pain and swelling. The symptoms had developed over the past month. On review of systems, the patient noted fatigue, malaise, and dull abdominal pain. She also described easy fatigability, exertional dyspnea, and a dry cough. She had lost 2 kg during this time.

Family history was notable for recent diagnosis of systemic brucellosis in patient's brother and sister. Her only medication was oral diclofenac tablet used to control join pain, which she had taken for 4 weeks.

On physical examination, the patient was alert and oriented, in mild respiratory distress, and complaining of shortness of breath. Temperature was 39°C, pulse rate 110/min, respiratory rate 28/min, and blood pressure 105/70 mmHg. Heart sounds were muffled. There was moderate diffuse abdominal tenderness with voluntary guarding. Both knees were swollen and erythematous with effusion but the right one bothered her the most. Her right ankle, both wrists, and right proximal interphalangeal (PIP) joint were similarly swollen, warm to touch, and erythematous. There were no rashes or mucosal lesions. Chest radiography showed an enlarged cardiac silhouette associated with bilateral pleural effusion.

Laboratory studies showed a white blood cell (WBC) count of 9,500/mm^3^ (72.2% neutrophils, 14.2% lymphocytes, and 13.6% mixed), hemoglobin 7.8 mg/dL, platelet count 395,000/mm^3^, aspartate aminotransferase (AST) 47 U/L, alanine aminotransferase (ALT) 54 U/L, alkaline phosphatase 1689 U/L, and gamma-glutamyltransferase (GGT) 103 U/L. Erythrocyte sedimentation rate was 104 and C-reactive protein 195 mg/L. Arthrocentesis of the right knee revealed a white blood cell count of 50,000 with 85% neutrophils. Abdominal sonography was unremarkable except for mild splenomegaly. According to the clinical syndrome, the following possible diagnoses were on the table: bacterial endocarditis, disseminated tuberculosis, systemic lupus erythematosus, adult onset still disease, brucellosis, and lymphoproliferative disorder associated with paraneoplastic autoimmune disease.

With regard to the differential diagnoses, laboratory tests including antinuclear antibodies (ANA) profile, serum C_3_ and C_4_ levels, Wright, 2-mercaptoethanol- (2ME-) Wright, and blood and synovial fluid stain and cultures, as well as echocardiography and high resolution chest computed tomography (CT) were performed. Patient started ceftriaxone plus vancomycin for the possible sepsis syndrome pending culture results. A high resolution chest CT revealed few peripheral patchy consolidations in the superior segment of right lower lobe and posterior segment of right upper lobe. Massive pericardial effusion was also noted. An echocardiogram reported large circumferential pericardial effusion with right ventricular diastolic collapse and exaggerated respiratory variation in mitral flow. No vegetation was detected. Pericardial window drainage was planned and patient was transferred to the operating room. After drainage, her respiratory distress resolved but she remained febrile despite receiving broad spectrum antibiotics.

Her initial blood cultures revealed penicillin-sensitive* Streptococcus pneumonia* in all culture bottles after 48 hours of incubation. Gram stain of the synovial aspirate showed no organisms and culture was negative at 1 week. Initial antibiotics stopped and high dose penicillin G started. The patient remained febrile after 96 hours of antibiotic treatment despite the fact that the blood cultures turned negative after 48 hours of starting antibiotics.

On the fifth day after hospital admission, the results of serologic tests showed high titers of antibodies to double-stranded DNA (over 500 U/mL), and low level of C_3_ 58 (75–135) mg/dL and C_4_ 9 (13–75) mg/dL. Serum immunoglobulin levels were in the normal range. A urinalysis showed 20 WBCs per high power field with no WBC or red blood cell (RBC) casts. Proteinuria was not detected. Other test results were unremarkable. On the basis of the available evidence, a diagnosis of systemic lupus erythematosus (SLE) was made, and treatment was started with high dose prednisone and hydroxychloroquine. She also planned for a 4-week course of intravenous antibiotic for possible right knee septic arthritis. Her symptoms improved gradually, fever stopped, and she was discharged from the hospital 30 days after the initial presentation.

## 2. Discussion

This is a rare case of systemic lupus erythematosus presenting with pneumococcal bacteremia. Based on the high resolution chest CT and the result of blood cultures, the bacteremia was assumed to be secondary to pneumococcal pneumonia. Because of the septic appearance of the patient and the result of blood cultures, pericardial effusion was initially thought to be bacterial, but it was later found to be sterile inflammatory fluid secondary to underlying connective-tissue disease.

Fever is a common finding during the disease activity in SLE patients. Eighty percent of SLE patients experience at least one episode of documented fever during the disease. Although patients with SLE are prone to infection, it is difficult to distinguish infection from active disease on clinical ground [1].

In several series, infection is reported as the leading cause of death in SLE patients [2]. Recently, Yurkovich et al. conducted a meta-analysis to determine the magnitude of risk from all-cause and cause-specific mortality in patients with SLE. Accordingly, SLE patients had a threefold increased risk of premature mortality from any cause compared with the general population. There was an almost 5-fold increase in the risk of death from infection in patients with SLE when compared with the general population [3]. Therapeutic, disease-related, and genetic factors including active (SLE) disease, long term disease damage, neutropenia, lymphopenia, hypocomplementemia, renal involvement, neuropsychiatric manifestations, and the use of glucocorticoids and other immunosuppressive drugs [4, 5] all have been reported to contribute to a lupus patient's increased susceptibility to infections [6, 7]. Hypocomplementemia also correlated with death from bacterial infections in SLE patients [2].

It has been noted that clinicians should consider the possibility of complement deficiencies in adult patients with severe and unusual infection due to encapsulated organisms, and the presence of hypocomplementemia should suggest the possibility of SLE, even in patients with no prior history of SLE [8]. In this situation, treatment of both diseases should be performed simultaneously.

Splenic dysfunction has been reported to occur in 5% of SLE patients and may be due to a variety of etiologies including circulating immune complexes and impairment of splenic circulation due to vasculitis or thrombotic events. SLE patients without functioning spleens are at especially high risk for severe infection, most commonly due to encapsulated bacteria [9]. To be cleared from the blood stream, encapsulated organisms require opsonisation with specific immunoglobulin and complement facilitating attachment to splenic macrophages via Fc and complement receptors. Thus, the defect in some patients with SLE could relate to a defect in opsonisation, Fc receptor function, or tissue macrophages. Few patients had irreversible defects with splenic atrophy and marked lymphocyte depletion found at necropsy. In others, the defect has been at least partially reversible and correlated best with disease activity and titer of circulating immune complexes [10].

The types of infections in SLE patients are similar to the general population [11]. Serious infectious complications develop in up to 50 percent of SLE patients [4, 6, 7]. SLE patients are more prone to develop common (pneumonia, urinary tract infection, cellulitis, and sepsis), chronic (tuberculosis), and opportunistic infections possibly due to inherit genetic and immunologic defects, but also due to the broad spectrum immunosuppressive agents that are part of therapy for severe manifestations of the disease [11]. In the study of Hsu et al., out of 51 patients with known history of SLE who were admitted to ICU, twenty-seven admissions (45 percent) were due to infectious diseases, including pneumonia with acute respiratory distress syndrome (ARDS) and sepsis of extrapulmonary origin. The infectious pathogens identified in SLE patients varied considerably and contained eleven cases of bacteremia (including* Pseudomonas aeruginosa, Salmonella*, and* Escherichia coli* that accounted for the cases of Gram-negative sepsis, whereas* Staphylococcus aureus, Staphylococcus epidermidis*, and* Streptococcus pneumoniae* were the major pathogens of Gram-positive sepsis), three cases with empyema (including one methicillin-resistant* S. aureus*, one* Streptococcus pneumoniae*, and one* Acinetobacter baumannii*), two patients with tuberculosis, and so forth [12].

Although infection is common and a leading cause of death in SLE [13], it is a rare initial presentation of the disease. SLE patients, especially those with hypocomplementemia, are at risk of infection with encapsulated bacteria. However, there are rare reports of severe invasive bacterial infection as the initial presentation of SLE [10, 14, 15]. It has been proposed that viral and bacterial infections may serve as environmental trigger for the development or exacerbation of SLE in the genetically predetermined individual [11]. Recent evidence has revealed that bacterial DNA can promote several of the autoimmune abnormalities observed in SLE, and a possible pathogenic role in the induction of SLE has been highlighted [16]. We found only few reports of severe bacterial infection as a new SLE presentation in prior literature (Table 1) [10, 14, 15, 17].

To most clinicians, the ability to distinguish an SLE flare from infection is a significant challenge. Because there is overlap between the organ involvement by SLE with that seen with infectious organisms, early recognition and appropriate treatment may be difficult. There are no early clinical and laboratory indicators of infection. However, several findings have been reported more frequently in patients with infection, including shaking chills, neutrophilic leucocytosis, and high CRP [1]. Elevated CRP levels in serum of SLE patients have been studied as a marker of disease activity versus the presence of coexistent infection. Although it has been suggested that elevated CRP levels can be used as a predictor of active infection in SLE patients with a high specificity, CRP levels during SLE flares can range widely in the number. On the other hand, fever may be observed in infected SLE patients even with nonelevated CRP levels [1, 18].

Procalcitonin (PCT) measurement may add to diagnostic accuracy in patients with systemic autoimmune diseases who present with a febrile illness, especially when highly sensitive PCT assays and specific PCT cutoff ranges are used in a predefined clinical setting. PCT levels are not significantly affected by renal function abnormalities or immunosuppressive agents. Therefore, significantly elevated PCT levels offer good specificity and sensitivity for systemic infection in patients with systemic autoimmune diseases, regardless of the use of corticosteroids or immunosuppressive agents [19, 20]. Recently, Serio et al. conducted a systematic review to examine the potential role of PCT for the discrimination between SLE flare and infection. They found no correlation between PCT levels and SLE disease activity. They also found that PCT levels detected during disease flares are lower than those observed during bacterial infections. Accordingly, they suggested that PCT can be used accurately in the early differentiation between bacterial infection and flare in febrile SLE patients [21]. However, PCT levels should not replace the necessary extensive diagnostic workup, which should include a thorough history and physical examination, combined with appropriate immunological, microbiological, radiological, and histological data [22]. Instead, elevated PCT levels (≥0.5 *μ*g/L) in SLE patients should always prompt a thorough search for sources of potential infection [21].

With regard to the fact that bacterial infections are the leading cause of morbidity in patients with SLE, it has been recommended to immunize all SLE patients with available bacterial vaccines to* Neisseria meningitidis* and* Streptococcus pneumonia* and to have a low threshold of suspicion for the diagnosis of disseminated neisserial or other encapsulated bacterial infections in SLE patients who are sick [23]. When some authors raised concerns regarding the safety and efficacy of immunizing persons who have connective-tissue disease, most studies found that pneumococcal vaccination of patients with SLE is safe and immunogenic in the majority of the patients [24].

In summary, we reported a rare case of invasive pneumococcal infection in a newly diagnosed SLE patient complicated with hypocomplementemia. Clinicians should be vigilant and mindful of the possible presence of underlying immune defect in patients who present with invasive pneumococcal disease. SLE is associated with a diverse spectrum of immune impairments including humoral defects that contribute to a lupus patient's increased susceptibility to infections with encapsulated bacteria [6, 7].

## Figures and Tables

**Table 1:** Cases of systemic lupus erythematosus presenting with an acute bacterial infection.

Authors	Number	Age	Sex	Clinical syndrome	Pathogen	Outcome
Petros and West [10]	1	18	F	Bacteraemia, disseminated intravascular coagulation, and multisystem organ failure	*S. pneumoniae*	Death

Elliott et al. [14]	2	6	M	Septic arthritis, bacteremia	*S. pneumoniae*	Survival

Segal et al. [15]	3	20	F	Bacteremia, multiple digital ischemia	*S. pneumoniae*	Survival
	4	50	F	Epiglottitis, bacteraemia, and multiorgan involvement	*H. influenzae*	Death

Charuvanij and Houghton [17]	5	5	F	Epiglottitis, bacteraemia	*H. influenzae*	Survival

Our report	6	14	F	Pneumonia, bacteremia	*S. pneumoniae*	Survival

## References

[1] Greenberg S. B. (2002). Infections in the immunocompromised rheumatologic patient. *Critical Care Clinics*.

[2] Hellmann D. B., Petri M., Whiting-O'Keefe Q. (1987). Fatal infections in systemic lupus erythematosus: the role of opportunistic organisms. *Medicine*.

[3] Yurkovich M., Vostretsova K., Chen W., Aviña-Zubieta J. A. (2014). Overall and cause-specific mortality in patients with systemic lupus erythematosus: a meta-analysis of observational studies. *Arthritis Care and Research*.

[4] Schur P. H., Gladman D. D., Basow D. S. (2011). Overview of the clinical manifestations of systemic lupus erythematosus in adults. *Up to Date*.

[5] Janwityanuchit S., Totemchokchyakarn K., Krachangwongchai K., Vatanasuk M. (1993). Infection in systemic lupus erythematosus. *Journal of the Medical Association of Thailand*.

[6] Paton N. I. (1997). Infections in systemic lupus erythematosus patients. *Annals of the Academy of Medicine, Singapore*.

[7] Fessler B. J. (2002). Infectious diseases in systemic lupus erythematosus: risk factors, management and prophylaxis. *Best Practice & Research Clinical Rheumatology*.

[8] Shaughnessy M. K., Williams D. N., Segal B. (2014). Severe infection with encapsulated bacteria as the initial presentation of systemic lupus erythematosus: two case reports and a review of the literature. *JMM Case Reports*.

[9] Santilli D., Govoni M., Prandini N., Rizzo N., Trotta F. (2003). Autosplenectomy and antiphospholipid antibodies in systemic lupus erythematosus: a pathogenetic relationship?. *Seminars in Arthritis and Rheumatism*.

[10] Petros D., West S. (1989). Overwhelming pneumococcal bacteraemia in systemic lupus erythematosus. *Annals of the Rheumatic Diseases*.

[11] Zandman-Goddard G., Shoenfeld Y. (2005). Infections and SLE. *Autoimmunity*.

[12] Hsu C.-L., Chen K.-Y., Yeh P.-S. (2005). Outcome and prognostic factors in critically ill patients with systemic lupus erythematosus: a retrospective study. *Critical Care*.

[13] Petri M. (1998). Infection in systemic lupus erythematosus. *Rheumatic Disease Clinics of North America*.

[14] Elliott J. A., Copeman A., Davies K. (2011). Pneumococcal sepsis as the first presentation of systemic lupus erythematosus (SLE) in a six year old boy. *Archives of Disease in Childhood*.

[15] Segal B., Williams D. N., Shaughnessy M. K. (2014). Severe infection with encapsulated bacteria as the initial presentation of systemic lupus erythematosus: two case reports and a review of the literature. *JMM Case Reports*.

[16] Tomita H., Yamada M., Sekigawa I., Yoshiike T., Iida N., Hashimoto H. (2003). Systemic lupus erythematosus-like autoimmune abnormalities induced by bacterial infection. *Clinical and Experimental Rheumatology*.

[17] Charuvanij S., Houghton K. M. (2009). Acute epiglottitis as the initial presentation of pediatric Systemic Lupus Erythematosus. *Pediatric Rheumatology*.

[18] Firooz N., Albert D. A., Wallace D. J., Ishimori M., Berel D., Weisman M. H. (2011). High-sensitivity C-reactive protein and erythrocyte sedimentation rate in systemic lupus erythematosus. *Lupus*.

[19] Quintana G., Medina Y. F., Rojas C. (2008). The use of procalcitonin determinations in evaluation of systemic lupus erythematosus. *Journal of Clinical Rheumatology*.

[20] Bador K. M., Intan S., Hussin S., Gafor A. H. (2012). Serum procalcitonin has negative predictive value for bacterial infection in active systemic lupus erythematosus. *Lupus*.

[21] Serio I., Arnaud L., Mathian A., Hausfater P., Amoura Z. (2014). Can procalcitonin be used to distinguish between disease flare and infection in patients with systemic lupus erythematosus: a systematic literature review. *Clinical Rheumatology*.

[22] Buhaescu I., Yood R. A., Izzedine H. (2010). Serum procalcitonin in systemic autoimmune diseases—where are we now?. *Seminars in Arthritis and Rheumatism*.

[23] Mitchell S. R., Nguyen P. Q., Katz P. (1990). Increased risk of neisserial infections in systemic lupus erythematosus. *Seminars in Arthritis & Rheumatism*.

[24] Elkayam O., Paran D., Caspi D. (2002). Immunogenicity and safety of pneumococcal vaccination in patients with rheumatoid arthritis or systemic lupus erythematosus. *Clinical Infectious Diseases*.

